# Effects of spirulina and wakame consumption on intestinal cholesterol absorption and serum lipid concentrations in non-hypercholesterolemic adult men and women

**DOI:** 10.1007/s00394-019-02073-7

**Published:** 2019-08-06

**Authors:** José J. van den Driessche, Jogchum Plat, Maurice C. J. M. Konings, Ronald P. Mensink

**Affiliations:** Department of Nutrition and Movement Sciences, NUTRIM School for Nutrition and Translational Research in Metabolism, Maastricht University Medical Center+ (MUMC+), P.O. Box 616, 6200 MD Maastricht, The Netherlands

**Keywords:** Spirulina, Wakame, Cholesterol, Intestinal cholesterol absorption, Humans

## Abstract

**Purpose:**

Consumption of the algae spirulina (*Arthrospira platensis or maxima)* and wakame (*Undaria pinnatifida*) has been shown to lower LDL cholesterol concentrations in animals and humans, possibly due to the inhibition of intestinal cholesterol absorption. This mechanism, however, has never been investigated in humans. Therefore, we examined in non-hypercholesterolemic men and women the effects of spirulina and wakame consumption on serum markers for intestinal cholesterol absorption.

**Methods:**

Thirty-five healthy men and women without hypercholesterolemia consumed in a random order daily 4.8 g spirulina, wakame or placebo for 17 days, separated by 14-day washouts. After 17 days, serum cholesterol-standardized campesterol, sitosterol and cholestanol, and lathosterol concentrations were measured as markers for intestinal cholesterol absorption and cholesterol synthesis, respectively. Concentrations of serum total cholesterol, LDL and HDL cholesterol, triacylglycerol, and plasma glucose, and blood pressure were measured as well.

**Results:**

Compared with placebo, spirulina or wakame did not affect serum cholesterol-standardized campesterol (CI − 0.23 to 0.10 μmol/mmol, *P* = 0.435 and CI − 0.14 to 0.19 μmol/mmol, *P* = 0.729, respectively), sitosterol (*P* = 0.314 and *P* = 0.112), cholestanol (*P* = 0.610 and *P* = 0.809), or lathosterol (*P* = 0.388 and *P* = 0.102) concentrations. In addition, serum lipid and plasma glucose concentrations, and blood pressure were not changed.

**Conclusions:**

Daily consumption of 4.8 g spirulina or wakame for 17 days did not affect plasma markers for intestinal cholesterol absorption or cholesterol synthesis in non-hypercholesterolemic men and women. Serum lipid and glucose concentrations, and blood pressure were also not altered.

**Electronic supplementary material:**

The online version of this article (10.1007/s00394-019-02073-7) contains supplementary material, which is available to authorized users.

## Introduction

Lowering serum LDL cholesterol (LDL-C) concentrations is a well-established strategy to reduce cardiovascular disease (CVD) risk [1]. This can be realized via inhibiting intestinal cholesterol absorption or suppressing endogenous cholesterol synthesis [2]. In this respect, not only drugs, but also diet plays an important role. Proven examples of natural compounds or foods affecting cholesterol absorption or synthesis include plant sterols and stanols, fibers, and red yeast rice [3, 4]. However, also other foods, such as algae, may contain bioactive components that lower serum LDL-C concentrations.

Consumption of algae has gained popularity in the Western world over the past few years, due to their postulated beneficial effects on CVD risk [5, 6]. Spirulina (*Arthrospira platensis* or *maxima*), belonging to the family of cyanobacteria, is a microalga containing high amounts of proteins, vitamins and light-harvesting structures such as C-phycocyanin [7]. Animal [8, 9] and several—but not all—human trials [10–15] have suggested that spirulina lowers serum total cholesterol (TC) and / or LDL-C concentrations. Studies in rats have now suggested that inhibition of intestinal cholesterol absorption could be the mechanism underlying the LDL-C reduction [9]. Wakame (*Undaria pinnatifida*) is one of the most-consumed macroalgae worldwide [16]. Constituents in wakame include the carotenoid fucoxanthin and fucoidan, a polysaccharide found in brown algae [17]. A limited number of studies have evaluated the cholesterol-lowering effects of wakame. Results from studies in rats on the effects of wakame [18–20] or its extract fucoxanthin [21] showed reductions in serum TC or LDL-C concentrations. On the other hand, three human studies did not show cholesterol-lowering effects of wakame [22–24], whereas a trial using fucoidan extracts from wakame did [25]. Again, inhibition of intestinal cholesterol absorption has been suggested as the underlying mechanism [19, 21].

Taken together, there is evidence both from human and animal studies that spirulina and wakame lower LDL-C concentrations, possibly by the inhibition of intestinal cholesterol absorption. However, this mechanism has never been examined in humans. Therefore, the aim of the present study was to evaluate in healthy, non-hypercholesterolemic men and women effects of spirulina and wakame consumption on markers for intestinal cholesterol absorption and endogenous cholesterol synthesis, and on serum lipid concentrations. Effects on glucose concentrations and blood pressure, as additional CVD risk markers [26], were studied as well.

## Subjects and methods

### Study population

Thirty-six apparently healthy men and women were recruited via online advertisements, posters in university and hospital buildings, and among subjects who had already participated in earlier studies within our department. Subjects were eligible for participation if they met the following criteria: aged between 18 and 70 years, BMI between 18 and 30 kg/m^2^, non-smoking, no use of medication or food supplements known to affect lipid or glucose metabolism or blood pressure, no conditions that might interfere with study outcomes, stable body weight (≤ 3 kg weight loss or gain in the past 3 months), no participation in another biomedical trial during the past month, and no abuse of drugs or alcohol. During a screening visit, fasting blood samples were taken to exclude subjects with elevated serum TC (≥ 8.0 mmol/L), serum triacylglycerol (≥ 4.5 mmol/L) or plasma glucose (≥ 7.0 mmol/L) concentrations. Furthermore, weight and height were measured for the determination of BMI. All subjects signed informed consent before the screening visit. This study was approved by the medical ethical committee of Maastricht University Medical Centre+ (MUMC+ ) and registered at clinicaltrials.gov as NCT03380611.

### Study design and intervention products

The study had a randomized, placebo-controlled, double-blind crossover design with three intervention periods of 17 days each, separated by washout periods of at least 14 days. Subjects were randomly assigned to one of the six possible treatment sequences for spirulina, wakame or placebo consumption. During the 17-day intervention periods, subjects consumed daily 12 capsules, each containing either 400 mg spirulina (Flora Health, Burnaby, Canada), 400 mg wakame (Swanson Health, Fargo, North Dakota, USA), or 400 mg microcrystalline cellulose (Radboud UMC, Nijmegen, The Netherlands). Thus, in total 4.8 g spirulina, 4.8 g wakame or placebo had to be consumed daily. This dose was used since it approximates the average dosage used in former studies with spirulina and wakame [22–24, 27]. Sterol composition of the spirulina and wakame capsules was measured using gas chromatography– flame ionization detection (GC–FID) by Bonn University (Supplemental Table 1). All capsules were different in appearance and subjects were not informed about the content of the capsules. At the start of each intervention period, capsules were provided in sachets labeled with A, B or C to blind the investigator. Subjects were instructed to take four capsules directly after breakfast, lunch and dinner. Empty sachets and unused capsules had to be returned and counted as a measure of compliance. Two weeks before the start and during the study, subjects were asked to abstain from foods and products containing algae, such as sushi or seaweed salads.

Subjects visited the university at the start (day 0) and twice at the end of each intervention period (days 14 and 17). They were asked to abstain from alcohol consumption and exercise the day preceding the visits. At each visit, fasting blood samples were taken by venipuncture after an overnight fast of at least 12 h. In addition, blood pressure and body weight were measured. At the end of each intervention period, subjects were asked to complete a validated food frequency questionnaire to assess food intake over the past 2 weeks. Energy and nutrient intakes were calculated using the Dutch food composition table (NEVO). Throughout the study, subjects were asked not to change their diets and physical activity patterns and were instructed to record daily any changes in health status and their potential alcohol consumption in a study diary.

### Blood sampling and analysis

Blood was drawn into serum and sodium fluoride (NaF)-containing tubes (Becton, Dickinson and Company, Franklin Lakes, NJ, USA) at each visit. Serum separator tubes were allowed to clot at room temperature for 30–60 min after withdrawal. Next, the tubes were centrifuged at 1300×*g* for 15 min at 21 °C to prepare serum. NaF-containing vacutainer tubes were placed on ice immediately after withdrawal and centrifuged at 4 °C for 15 min at 1300×*g* to prepare NaF plasma. Serum and NaF plasma samples were directly frozen in liquid nitrogen and stored at − 80 °C until analysis. For all analysis, all samples from one subject were analyzed in the same analytical run.

Serum plant sterol (campesterol, sitosterol) concentrations, cholestanol, and concentrations of the cholesterol precursor lathosterol were measured in samples collected at the end of each intervention period (days 14 and 17) by GC–FID as previously described [28]. Values were standardized for total cholesterol concentrations as measured by GC–FID, and expressed as μmol/mmol total cholesterol.

Serum TC concentrations (CHOD-PAP method; Roche Diagnostics System, Mannheim, Germany), HDL cholesterol (HDL-C) concentrations (precipitation method followed by CHOD-PAP method; Roche Diagnostics System), triacylglycerol concentrations corrected for free glycerol (GPO-Trinder, Sigma Diagnostics, St Louis, USA), high-sensitivity C-reactive protein (hsCRP) concentrations (immunoturbidimetric assay, Horiba ABX, Montpellier, France) and plasma glucose concentrations (Horiba ABX) were measured in all samples. LDL-C concentrations were calculated using the Friedewald formula [29].

### Blood pressure measurements

Systolic and diastolic blood pressure was determined after a 5-minute rest in seated position during every visit (Omron M7, Omron Healthcare Co., Ltd., Kyoto, Japan). Four measurements were performed. The first measurement was discarded and the last three measurements were averaged for data analyses.

### Statistics

It was estimated that a sample size of 33 subjects was needed to detect a true difference of 0.24 μmol/mmol in cholesterol-standardized campesterol concentrations with a power of 80% and a within-subject variability of 0.47 μmol/mmol [30, 31]. This effect size was chosen since earlier studies from our group showed comparable effects using plant stanol supplementation [30–32]. As the anticipated dropout rate was 10%, 36 subjects were recruited.

All results are presented as means ± SDs. Values at the end of the three periods (days 14 and 17) were averaged for all parameters. A priori, it was decided that comparisons would only be made between the spirulina and control conditions, and between the wakame and control conditions, and not between the spirulina and wakame conditions. Differences in end-of-intervention values between spirulina or wakame and control conditions were compared using linear mixed models with subject as random factor, and treatment and period as fixed factors. Differences in end-of-intervention hsCRP concentrations were compared using the non-parametric Friedman test. *P* values < 0.05 were considered to be statistically significant. The interaction term treatment * period was used to test for carry-over effects with linear mixed models. However, this interaction term never reached statistical significance and was, therefore, removed from all models. Data were analyzed separately for men and women and baseline TC concentrations above and below 5.0 mmol/L, but this did not change the conclusions. The spirulina and wakame conditions were each compared with the placebo condition using post hoc tests. To correct for multiple comparisons, P values < 0.025 were then considered statistically significant. Statistical analyses were performed using SPSS 25.0 for Mac (IBM Corp., Armonk, NY, USA).

## Results

### Subjects and compliance

**Fig. 1:**
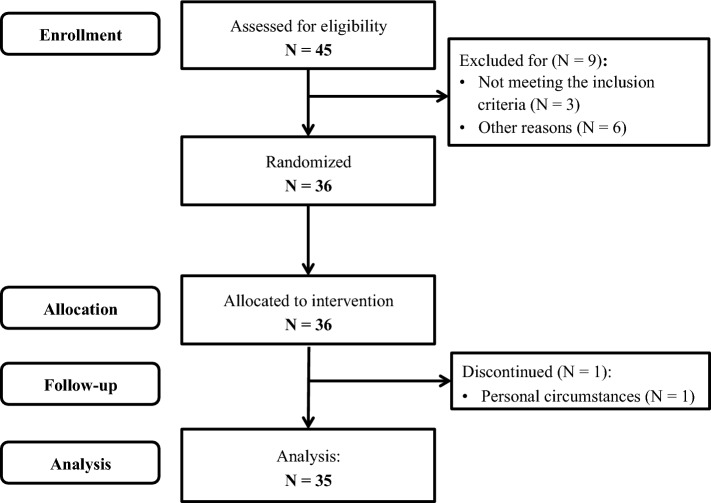
Flowchart of participants throughout the study

Thirty-six subjects started the intervention and one subject dropped out due to personal reasons (Fig. 1). In the end, 35 subjects (15 men and 20 women) completed the trial and were included in the statistical analyses. LDL-C data for one subject could not be calculated due to triacylglycerol concentrations above the 4.52 mmol/L threshold for reliable use of the Friedewald formula [29]. Baseline characteristics of the 35 subjects that completed the trial are shown in Table 1. Changes in weight of the subjects did not differ between the spirulina (− 0.2 ± 0.7 kg), wakame (− 0.1 ± 0.6 kg) and placebo periods (− 0.2 ± 0.6 kg; *P* = 0.925 for treatment effect). Serum hsCRP concentrations also did not differ between the three intervention periods (*P* = 0.450). Overall compliance was 99% (98.2–99.5%) based on capsule count.

**Table 1:** Baseline characteristics of men and women who completed the study (*N* = 35)

	Mean ± SD
Men/women, *n*	15/20
Age (years)	40.2 ± 19.6
BMI (kg/m^2^)	24.7 ± 2.7
Weight (kg)	71.9 ± 12.1
Total cholesterol (mmol/L)	4.9 ± 1.1
HDL cholesterol (mmol/L)	1.7 ± 0.5
LDL cholesterol (mmol/L)	2.7 ± 1.0
Triacylglycerol (mmol/L)	1.1 ± 0.6
Glucose (mmol/L)	5.2 ± 1.0

### Dietary intake

Average daily intakes of energy and the macronutrients did not differ between the three intervention periods (Table 2**)**. In addition, cholesterol and fiber intakes were also not different.

**Table 2:** Dietary intake as assessed with food frequency questionnaires after spirulina, wakame and placebo intake

	Spirulina	Wakame	Placebo
Energy (MJ/day)	9.1 ± 2.4	8.7 ± 2.8	9.2 ± 2.9
Fat (energy %)	37.6 ± 7.6	37.9 ± 7.6	39.2 ± 8.6
SFA	12.7 ± 3.9	12.7 ± 3.3	13.2 ± 3.4
MUFA	14.1 ± 3.5	14.4 ± 3.4	14.9 ± 4.0
PUFA	7.3 ± 2.3	7.2 ± 2.3	7.6 ± 2.9
Protein (energy %)	16.3 ± 3.1	16.7 ± 3.3	16.4 ± 3.3
Carbohydrates (energy %)	40.5 ± 7.1	39.9 ± 8.0	39.1 ± 8.7
Alcohol (energy %)	3.0 ± 2.6	3.0 ± 2.2	2.8 ± 2.4
Fiber (g/day)	26.3 ± 5.5	24.4 ± 7.9	24.2 ± 5.6
Cholesterol (mg/day)	238 ± 113	224 ± 124	256 ± 160

*SFA* saturated fatty acids, *MUFA* monounsaturated fatty acids, *PUFA* polyunsaturated fatty acids

### Serum plant sterols, cholestanol and lathosterol concentrations

Concentrations of cholesterol-standardized serum campesterol, sitosterol and cholestanol, markers for intestinal cholesterol absorption, did not differ between the spirulina and placebo conditions (*P* = 0.435, *P* = 0.314, *P* = 0.610, respectively), or the wakame and placebo conditions (*P* = 0.729, *P* = 0.112, *P* = 0.809, respectively; Table 3). Serum cholesterol-standardized lathosterol concentrations, a marker for cholesterol synthesis, did also not differ between the spirulina or wakame and placebo conditions (*P* = 0.388 and *P* = 0.102, respectively).

**Table 3:** Serum cholesterol-standardized concentrations of plant sterols and lathosterol, and lipid concentrations after spirulina, wakame and placebo intake (*N* = 35)

	Spirulina	Wakame	Placebo	Estimated difference (versus placebo)^a^	
				Spirulina	Wakame
Campesterol (μmol/mmol)	2.60 ± 1.11	2.69 ± 1.11	2.66 ± 0.99	− 0.07 (− 0.23 to 0.10)	0.03 (− 0.14 to 0.19)
Sitosterol (μmol/mmol)	2.25 ± 0.76	2.42 ± 0.75	2.32 ± 0.67	− 0.07 (− 0.19 to 0.06)	0.10 (− 0.03 to 0.23)
Cholestanol (μmol/mmol)	1.51 ± 0.33	1.52 ± 0.31	1.52 ± 0.33	− 0.01 (− 0.06 to 0.03)	− 0.01 (− 0.05 to 0.04)
Lathosterol (μmol/mmol)	1.58 ± 0.52	1.62 ± 0.50	1.54 ± 0.50	0.04 (− 0.05 to 0.13)	0.08 (− 0.02 to 0.17)
Total cholesterol (mmol/L)	4.75 ± 1.00	4.84 ± 1.02	4.81 ± 1.09	− 0.06 (− 0.22 to 0.10)	0.03 (− 0.13 to 0.18)
LDL cholesterol (mmol/L)b	2.75 ± 0.97	2.78 ± 1.04	2.77 ± 1.07	− 0.03 (− 0.15 to 0.10)	0.01 (− 0.12 to 0.13)
HDL cholesterol (mmol/L)	1.52 ± 0.43	1.56 ± 0.42	1.56 ± 0.49	− 0.04 (− 0.11 to 0.03)	0.00 (− 0.07 to 0.07)
Triacylglycerol (mmol/L)	1.09 ± 0.63	1.12 ± 0.80	1.06 ± 0.61	0.02 (− 0.09 to 0.14)	0.06 (− 0.06 to 0.18)

^a^Estimated difference and 95% confidence interval (CI), based on estimated marginal means obtained with linear mixed models

^b^*N* = 34 for LDL cholesterol concentrations

### Serum lipids

Serum lipid concentrations are shown in Table 3. No differences were found between the spirulina and placebo conditions for serum total cholesterol (*P* = 0.443), LDL-C (*P* = 0.677), HDL-C (*P* = 0.273) and triacylglycerol concentrations (*P* = 0.684). Serum total cholesterol (*P* = 0.749), LDL-C (*P* = 0.902), HDL-C (*P* = 0.937), and triacylglycerol concentrations (*P* = 0.302) did also not differ between the wakame and placebo conditions. When subjects were divided into the 50% highest and 50% lowest ‘cholesterol absorbers’ based on the median lathosterol to campesterol ratio [33], still no differences were found between the spirulina or wakame versus the control conditions within the two subgroups (Supplemental Table 2).

### Glucose concentrations and blood pressure

No differences were found between the spirulina and placebo conditions for plasma glucose concentrations (*P* = 0.375), as well as between the wakame and placebo conditions (*P* = 0.373). Systolic and diastolic blood pressure did also not differ between the spirulina and placebo (*P* = 0.651 and *P* = 0.550, respectively; Table 4), or the wakame and placebo conditions (*P* = 0.620 and *P* = 0.677, respectively).

**Table 4:** Plasma glucose concentrations, and systolic and diastolic blood pressures after spirulina, wakame and placebo consumption (*N* = 35)

	Spirulina	Wakame	Placebo	Estimated difference (versus placebo) ^a^	
				Spirulina	Wakame
Glucose (mmol/L)	5.27 ± 0.37	5.27 ± 0.39	5.23 ± 0.37	0.04 (− 0.04 to 0.11)	0.04 (− 0.04 to 0.11)
Systolic blood pressure (mmHg)	113.9 ± 13.7	114.1 ± 14.3	114.4 ± 14.5	− 0.5 (− 2.8 to 1.8)	− 0.6 (− 2.9 to 1.7)
Diastolic blood pressure (mmHg)	75.4 ± 9.4	75.3 ± 9.3	74.9 ± 9.5	0.5 (− 1.1 to 2.1)	0.3 (− 1.3 to 1.9)

^a^Estimated difference and 95% confidence interval (CI), based on estimated marginal means obtained with linear mixed models

## Discussion

In this placebo-controlled double-blind intervention study, daily consumption of 4.8 g spirulina or 4.8 g wakame for 17 days did not affect markers for intestinal cholesterol absorption and endogenous cholesterol synthesis in non-hypercholesterolemic healthy men and women. In agreement, serum lipid concentrations were also not affected. Also, no effects on plasma glucose concentrations and blood pressure were observed.

Animal studies have suggested that spirulina and wakame consumption inhibits intestinal cholesterol absorption. In male Wistar rats, a spirulina concentrate increased fecal steroid content with a concomitant decrease in LDL-C concentrations [9]. In two in vitro experiments, a spirulina concentrate decreased micellar solubility of cholesterol and suppressed cholesterol absorption in Caco-2 cells. Similarly, supplementation with wakame or a wakame extract increased fecal cholesterol excretion in male Wistar rats [19] and C57BL/6J mice on a high-fat diet [21], again suggesting the inhibition of intestinal cholesterol absorption. However, since serum cholesterol-standardized campesterol, sitosterol and cholestanol concentrations were not changed, our results do not suggest that these two algae did have an effect on intestinal cholesterol absorption in humans. In addition, cholesterol-standardized lathosterol concentrations, a marker reflecting endogenous cholesterol synthesis, were not altered. The use of serum non-cholesterol sterol and stanol concentrations as markers for intestinal cholesterol absorption and endogenous cholesterol synthesis has been well validated [34]. Yet, when the intake of these sterols changes, plasma levels do not reflect cholesterol absorption anymore. However, levels of sterols in the algae were very low (Supplemental Table 1) and, therefore, did not affect the validity of plasma plant sterols as markers for intestinal cholesterol absorption. In addition, cholestanol was not present in the algae and the observation that serum cholestanol concentrations were not affected confirmed the lack of an effect on intestinal cholesterol absorption.

As expected by the lack of effects on intestinal cholesterol absorption and endogenous cholesterol synthesis, serum TC or LDL-C concentrations were also unchanged. This contrasts findings from a recent meta-analysis, including 10 RCTs with 12 treatment arms and more than 700 subjects, evaluating the effects of spirulina consumption on serum lipid concentrations [27]. Decreases of − 1.00 mmol/L and − 0.91 mmol/L were reported for TC and LDL-C respectively. These effects are large for a dietary intervention and are in the range of those achieved with drugs [35]. Although the present study was not primarily powered on changes in LDL-C, post hoc calculations showed that the statistical power of our study was close to 100% to pick up such an effect. In the same meta-analysis, a decrease in triacylglycerol concentrations was found, whereas those of HDL-C were not significantly changed. However, 5 of the 10 RCTs measuring serum lipid concentrations were not blinded, since the control groups received no placebo capsules or tablets [10, 12–14, 36]. In fact, subgroup analysis revealed that TC, LDL-C, TAG concentrations decreased and those of HDL-C increased in the trials with a no-intervention control group. When a placebo group was included, only TC concentrations decreased. Differences in dose, duration of the intervention and study populations are factors to explore in trying to explain discrepancies in results.

Intake of spirulina differed largely between the 10 trials included in the meta-analysis ranging from 1 to 19 g daily, with a median intake of 2 g. Subgroup analysis suggested that lipid-lowering effects were found with consumption of 2 g or more, whereas no significant effects were found with intakes less than 2 g a day. As our daily dose of 4.8 g is clearly above this median intake of 2 g, it is unlikely that differences in spirulina dosage could explain the lack of effects.

The duration of our intervention was shorter compared to earlier trials. The median intervention duration in the meta-analyses of Huang and colleagues was 12 weeks. Subgroup analysis revealed that significant changes in lipid concentrations were only found in the 7 RCTs lasting 12 weeks or longer, but only three of them used placebos instead of a no-intervention control group. It is not likely that our shorter study duration can explain the lack of effect on LDL-C, as LDL-C concentrations reach a new steady state within 2 weeks when intestinal cholesterol absorption is inhibited by dietary components or drugs [37, 38].

Our study population also varied from those of other studies. Spirulina lowered TC, LDL-C and TAG, but not HDL-C concentrations in type II diabetics [11, 39] and children with the nephrotic syndrome [14]. On the other hand, two other studies in type II diabetics only reported TAG-lowering effects and no effects on TC, LDL-C and HDL-C concentrations [10, 40]. Lipid concentrations were all improved in ischemic heart disease patients with hypercholesterolemia, whereas no effects on any of the lipid parameters were seen in obese subjects [15]. In HIV patients [12] and hypertensive subjects [41], TC and LDL-C concentrations decreased and those of HDL-C increased. In elderly, only TC concentrations were decreased [42]. Overall, heterogeneity between studies was large and there was no evidence that some populations were more responsive than others. It is, therefore, not likely that the effects of spirulina consumption are only evident in subjects with increased baseline total cholesterol concentrations and not in our healthy, non-hypercholesterolemic population. Also, studies with plant sterols and stanols have demonstrated LDL-C lowering effects via inhibition of intestinal cholesterol absorption in non-hypercholesterolemic subjects [32, 37]. In conclusion, there is no clear reason why our results do not support the results of the meta-analysis of Huang et al. [27]. Possibly, lack of blinding of some of the earlier studies may have biased outcomes.

Results of human trials investigating the effect of wakame consumption on TC or LDL-C concentrations are more in line with our results in non-hypercholesterolemic subjects. No effects were found in hypertensive subjects [22], subjects with the metabolic syndrome [23], and HIV patients [24]. In former trials, daily intakes ranged between 4 and 6 g, which is comparable to the intake used in our study. In one study, 500 mg of fucoidan extracted from brown seaweed lowered LDL-C concentrations in overweight and obese subjects [25]. However, this amount of fucoidan is present in 13–46 g of wakame [43], which is much higher than the amount of 4.8 g provided in our and the other studies. Whether the wakame extract fucoidan truly lowers LDL-C warrants further study.

Glucose concentrations and blood pressure were assessed as additional markers for CVD risk, but were not changed by spirulina or wakame consumption. A meta-analysis including eight RCTs suggested glucose-lowering effects of spirulina consumption [27], which is in contrast with our results. No subgroup analyses were performed. Of the 8 studies included, 4 were certainly not blinded. Decreases in fasting glucose concentrations were observed in studies with type II diabetics [11, 36, 39], HIV patients [44], and hypertensive subjects [41]. However, in two other trials with type II diabetics [10, 40] and a trial with children with the nephrotic syndrome [14], glucose concentrations were not affected. None of the trials with wakame reported effects on glucose concentrations [23, 24]. Diastolic blood pressure was also significantly lowered after spirulina consumption in the recent meta-analysis [34], whereas systolic blood pressure was not affected. Three studies were included in the analysis, of which two were blinded. However, in only one individual trial with hypertensive subjects, spirulina consumption significantly affected diastolic blood pressure [45]. Wakame consumption did affect systolic and diastolic blood pressure in hypertensive subjects [22] and systolic blood pressure in subjects with the metabolic syndrome [23]. In the latter trial, effects were only present in a hypertensive subgroup. Thus, it may be that algae consumption only lowers blood pressure in subjects with increased baseline blood pressure levels. Although this needs to be explored further, it might explain the lack of an effect on blood pressure in our trial with non-hypertensive subjects.

To conclude, our study indicates that consuming 4.8 g/day spirulina or wakame for 17 days does not inhibit intestinal cholesterol absorption in non-hypercholesterolemic men and women, nor does it affect lipid profiles. In addition, blood pressure and glucose concentrations were not affected by spirulina or wakame consumption.

## Electronic supplementary material

Below is the link to the electronic supplementary material.

Supplementary file1 (PDF 82 kb)
