# Supplementary material for: Effects of spirulina and wakame consumption on intestinal cholesterol absorption and serum lipid concentrations in non-hypercholesterolemic adult men and women
Source: Eur J Nutr. 2019 Aug 6;59(5):2229–36. doi: 10.1007/s00394-019-02073-7 (PMC7351869; doi:10.1007/s00394-019-02073-7)
Supplement: Supplementary file 1 — Supplementary file1 (PDF 82 kb) [file 394_2019_2073_MOESM1_ESM.pdf]

Effects of spirulina and wakame consumption on intestinal cholesterol absorption and serum lipid concentrations in non-hypercholesterolemic men and women

- Supplemental Materials -

José J van den Driessche, Jogchum Plat, Maurice Konings, Ronald P Mensink

Department of Nutrition and Movement Sciences, NUTRIM School for Nutrition and Translational Research in Metabolism, Maastricht University Medical Center+ (MUMC+), Maastricht, the Netherlands.

Corresponding author:
Ms. José J. van den Driessche
Email: j.vandendriessche@maastrichtuniversity.nl

SUPPLEMENTAL TABLES

Supplemental table 1: Sterol composition of the spirulina and wakame supplements as determined by gas-chromatography flame-ionization-detection (GC-FID) by Bonn University

	**Spirulina**		**Wakame**	
	**ng/mg**	**µg/day**	**ng/mg**	**µg/day**
Cholesterol	7.8	37.4	-	-
24-methyl cholesterol	7.7	37.0	65.1	312.5
Campesterol	4.9	23.5	-	-
Sitosterol	19.3	92.6	-	-
Stigmasterol	11.5	55.2	-	-
Fucosterol	-		476.6	2287.7
24 R/S saringosterol	-		17.4	83.5

Supplemental table 2: Lipid concentrations after spirulina, wakame and placebo intake in the 50% highest absorbers (N = 17) and the 50% lowest absorbers (N = 18)

	Highest cholesterol absorbers				Lowest cholesterol absorbers			
	Spirulina	Wakame	Placebo	P [a]	Spirulina	Wakame	Placebo	P [a]
Total cholesterol (mmol/L)	4.28 ± 0.76	4.42 ± 0.89	4.48 ± 0.84	0.269	5.13 ± 1.04	5.24 ± 1.02	5.11 ± 1.26	0.645
LDL cholesterol (mmol/L) [b]	2.24 ± 0.57	2.33 ± 0.75	2.34 ± 0.73	0.575	3.26 ± 0.83	3.24 ± 0.92	3.21 ± 1.01	0.871
HDL cholesterol (mmol/L)	1.65 ± 0.42	1.65 ± 0.43	1.72 ± 0.51	0.429	1.43 ± 0.39	1.52 ± 0.36	1.42 ± 0.41	0.119
Triacylglycerol (mmol/L)	0.86 ± 0.34	0.96 ± 0.46	0.93 ± 0.35	0.422	1.16 ± 0.46	1.07 ± 0.48	1.03 ± 0.39	0.488

[a] P-value for the treatment effect

[b] N = 17 in the 'lowest cholesterol absorbers' for LDL cholesterol
